# Co-creation in primary healthcare: a qualitative study of collaboration between welfare technologists and healthcare professionals

**DOI:** 10.1108/JHOM-04-2025-0223

**Published:** 2026-01-30

**Authors:** Inger Lise Teig, Ragnhild Gjerstad-Sørensen, Brita Gjerstad

**Affiliations:** Department of Global Public Health and Primary Care, University of Bergen, Bergen, Norway; Department of Health and Social Sciences, NORCE Norwegian Research Centre AS, Bergen, Norway; Faculty of Social Sciences, University of Stavanger, Stavanger, Norway

**Keywords:** Co-creation, Healthcare professionals, Primary healthcare, Qualitative study, Technologists, Welfare technology

## Abstract

**Purpose:**

This study contributes to the growing body of research on co-creation in primary healthcare by highlighting the complex and evolving relationship between municipalities and welfare technology providers. Drawing on the framework of “co-creation,” the study aims to illuminate the dynamic interplay between technologists and other stakeholders in shaping healthcare services.

**Design/methodology/approach:**

A qualitative approach utilising reflexive thematic analysis was employed. The study consisted of 17 semi-structured interviews with healthcare managers from three municipalities and technologists from four private welfare technology companies.

**Findings:**

In this study, three overarching themes were developed: (1) Welfare technologists enter the public sector arena from below, (2) Bridging sectors: The growing complexity of municipality–technologist collaboration, and (3) Skill expansion – an impact of emerging technologies. Our findings indicate that co-creation fosters balanced partnerships, with processes characterised by consensus and shared goals. Rather than passive recipients, municipal actors actively shape healthcare services. While welfare technologists are seen both as partners and vendors, municipalities maintain a strategic role in procurement and in aligning technological solutions with public service values.

**Research limitations/implications:**

One notable limitation is the exclusive focus on the provider side of healthcare services, omitting the perspective of patients and their relatives. This focus is justified by the underexplored role of technologists in health services research, despite their crucial influence on the valuation and implementation of technology. The participant selection process, which involved managers recommending additional participants, ensured relevance but may have introduced bias based on managerial attitudes. However, the data does not indicate significant bias, as participants expressed both positive and negative perspectives. A key strength of this study lies in its dual perspective, incorporating insights from both private-sector technologists and public healthcare managers and professionals. The analysis highlights the complexity of integrating welfare technology into primary healthcare systems. By employing a co-creation framework, the study sheds light on the distinct roles of co-creators and offers valuable insights into how these actors navigate the intersection of care work and technological development.

**Social implications:**

The increasing role of welfare technologists in healthcare raises important questions about control, autonomy, and the future governance of welfare technologies. It may reflect a broader shift in healthcare governance, where healthcare services are co-constructed through cooperation, mutual learning, and the balancing of technical possibilities with care values.

**Originality/value:**

A key strength of this study lies in its dual perspectives, incorporating insights from both private-sector technologists and public healthcare managers and professionals. It explores how technologists have positioned themselves as key actors in the primary healthcare sector and reveals that technologists strategically present themselves as well-informed about the sector’s challenges. This enables them to gain acceptance within municipalities, where they are recognised as important partners in shaping healthcare services.

## Introduction

An ageing population and limited resources are placing increasing pressure on healthcare systems worldwide. In response, technologies designed to support older adults in managing daily life independently – often referred to as gerontechnology, Active Assisted Living, or welfare technology – have gained traction as potential solutions (Søraa *et al*., 2021; Kiran, 2017). The Nordic healthcare system is characterised by high quality and early adopters of new treatments and technology (Brennan *et al*., 2015; Pojiltov and Mainela, 2025). Welfare technology, in particular, has become prominent, where it is being integrated into public healthcare systems with the promise of reducing costs and enabling elderly individuals to live independently at home (Hofmann, 2013; Aaen, 2021; Søraa *et al*., 2021). Trust in these technologies is substantial (Kjøllesdal, 2010; Lupton, 2014; Fugletveit and Sørhaug, 2023), and their perceived potential is deeply embedded in political strategies and the expansion of health technology markets (Aaen, 2021).

Healthcare innovation is shaped by many interrelated factors. Aging populations and shifting care needs are important considerations, and the adoption of digital technologies plays a critical role in transforming service delivery, improving efficiency, and enhancing patient engagement. The perspectives of stakeholders, such as healthcare professionals, patients, and private actors, are vital as these experiences and expectations influence how technologies are implemented and received.

These dynamics unfold differently across healthcare contexts, each presenting distinct challenges such as resource limitations, technological readiness, and evolving governance models. By viewing demographic trends, technological developments, and stakeholder engagement as interconnected factors, we gain a more comprehensive understanding of the drivers of change in contemporary healthcare systems.

Against this backdrop, our study asks: *How do technologists and healthcare professionals collaborate, and what consequences does this collaboration lead to?* These questions guide our exploration of co-creative processes in primary healthcare, focusing on how collaboration shapes the complex and evolving relationship between municipalities and welfare technology providers.

### Welfare technology in Norwegian healthcare

In Norway, welfare technology became central to health and care policies in 2011 through initiatives by the Ministry of Health and Care Services. This included technical devices, care services technologies, smart house solutions, and communication tools which are mainly intended for use in the residents’ homes and will also be partly used by them (NOU, 2011, p. 11). The political aim was, as it still is, to reduce healthcare costs by enabling the elderly to live at home rather than in institutional settings (Meld St. 15, 2017–2018; Meld St. 24, 2022–2023; NOU 2011, p. 11).

Norwegian authorities have encouraged welfare technologists, i.e. technologists who are developing technologies for independent living, through various support mechanisms, including tax incentives, research programs, and targeted funding. Correspondingly, municipalities, which are responsible for the primary health care, have received support for adopting new technologies through guidelines, recommendations, standardisations, evidence-based research, financial support for pilot projects, and the National Welfare Technology Program, a program that aimed to support development and implementation of welfare technology in primary healthcare services (Meld. St. 29 (2012–2013)). However, while municipalities are tasked with identifying needs and procuring welfare technology, there is less emphasis on the role of welfare technologists or on fostering mutual understanding between welfare technologists and healthcare providers.

### Challenges in collaboration and implementation

Procuring welfare technology demands substantial expertise, expertise that municipalities are often reported to lack (Dugstad *et al*., 2015). Many municipalities have limited knowledge of welfare technology, are unaware of available solutions, or lack a clear understanding of how specific tools function (Branstad, 2015). On the other hand, suppliers may demonstrate overconfidence in their products, sometimes failing to adequately consider the distinct needs of municipalities and their end users (Dugstad *et al*., 2015; Gjelsvik *et al*., 2016). This mismatch in knowledge and expectations contributes to challenges in the procurement processes and in collaboration between stakeholders.

The successful implementation of welfare technology can also be hindered by stakeholders’ lack of knowledge about each other’s fields. For instance, Salminen-Karlsson and Golat (2022) found that a health information system in a Swedish hospital failed to align with nurses’ holistic care routines. Similarly, Marchesoni *et al*. (2017) reported that the introduction of smartphones disrupted healthcare staff and created stress by imposing a rationality that conflicted with patient care priorities. Additionally, the use of technology in healthcare raises various ethical challenges. Hofmann (2013) highlighted that technology can reduce the relational interplay necessary to assess the level and severity of illness risk. Heinsch *et al*. (2021) noted that technology could convey suicidal ideations, socially unacceptable content, and possible child neglect, while also violating privacy (Laholt *et al*., 2019).

Innovation and the development of new solutions depend on the effective exchange of problems, knowledge, and ideas. However, collaboration can be challenged both by excessive similarity, limiting diversity of perspectives, and by significant differences, which may create barriers to mutual understanding and collaboration (Krogh *et al*., 2020). As Røiseland and Vabo (2016) argue, when actors in collaborative processes possess unequal access to knowledge or resources, there is a risk that more powerful stakeholders may dominate decision-making, thereby compromising the principles of equitable and inclusive collaboration (Røiseland and Vabo, 2016). This dynamic is particularly relevant in primary healthcare, where care and technology are often perceived as belonging to distinct spheres (Kjøllesdal, 2010), each shaped by different rationalities and cultures (Hofmann, 2013). In such contexts, technological rationality tends to dominate, raising concerns about how collaborative processes can remain inclusive and responsive to all stakeholders’ perspectives.

For years, public procurement regulations posed significant challenges, making transactions lengthy and cumbersome and offering limited opportunities for product testing (Gjelsvik *et al*., 2016). Technologists generally prefer direct communication with healthcare and technical staff to align solutions effectively; however, according to technologists, municipalities often handle procurement without involving technical personnel (Branstad, 2015). This results in varying levels of cooperation between municipalities and suppliers, ranging from close collaboration to distant interaction.

While recent adjustments to procurement regulations have aimed to simplify cooperation, significant difficulties persist (Sande *et al*., 2022). These regulations can inadvertently create distance between public and private actors, complicating efforts to foster co-creation and reducing the likelihood of achieving innovative and effective solutions (Torfing *et al*., 2019).

### Knowledge gaps in “welfare technology” implementation and stakeholder engagement

Despite its growing prominence, significant knowledge gaps persist regarding welfare technology’s implementation (Borg *et al*., 2022), and research on the processes of welfare technology development is limited (Aaen, 2021). Aljafari *et al*. (2024) recently documented that there is still limited research focusing specifically on “patient-engaging digital services” (i.e. electronic initiatives that involve patients in the process of healthcare service creation and delivery, cf. Agarwal *et al*., 2010). Policymakers’ optimism often contrasts with slower adoption rates, reflecting unresolved and conflicting priorities among stakeholders, including public providers, private companies, end-users, and policymakers (Greenhalgh *et al*., 2012; Aaen, 2021). Technologists are often seen as profit-driven actors (Pollock and Williams, 2009). However, their influence on healthcare professionals’ decisions and practices during technology adoption is significant (Pollock and Williams, 2010). They play a crucial role in shaping how technology is valued and implemented (Lehoux *et al*., 2017). Despite this, their role in the development of public healthcare remains underexplored in health services research.

### The study context and research aim

Elderly care in Norway is funded by taxes and mainly delivered through public services. While the central government holds overarching authority and supervises municipal practice, each municipality, regardless of size, serves as the main provider of welfare services across the country. Norwegian health policy strategies emphasise the role of technology in promoting individual independence, aiming to enhance individuals’ ability to manage their daily lives despite illness and social, psychological or physical impairment (NOU, 2011, p. 11).

Norway is likely more technology-friendly and technology-dependent than many other countries. This is evident from the fact that 99% of Norwegian households have internet access, and 96% of people aged 16–74 use it daily (www.ssb.no). However, among those aged 75–79, the corresponding figure for weekly internet use is 78% (www.ssb.no), indicating a risk of digital marginalisation among older adults (Rybalka *et al*., 2022). Compared to the rest of Europe, digital skills in Norway are generally high (Fjørtoft, 2017). As a wealthy nation, Norway also has greater financial capacity to implement technology effectively (Lange *et al*., 2018). The Norwegian government has demonstrated a strong commitment to digitalising its welfare system using welfare technology, aiming to harness the potential benefits of these advancements (Fugletveit and Sørhaug, 2023).

By addressing a notable gap in existing research, this article sheds light on the collaboration between welfare technologists and healthcare managers/professionals in the design and organisation of healthcare services. To understand these dynamics, qualitative semi-structured interviews were conducted with municipal healthcare professionals and managers, and welfare technology developers and suppliers. Using the framework of “co-creation”, the study highlights the dynamic interaction between welfare technologists and other stakeholders, offering insights into how collaborative processes shape the development of healthcare services.

## Theoretical framework: digital transformation and co-creation in healthcare

The digital transformation of healthcare has significantly reshaped service delivery across various settings, including hospitals, clinics, outpatient care, and local health services (Mimoso *et al*., 2024). This transformation is not solely driven by technological advancement but is also embedded in organisational, professional, and governance dynamics. Research on digital innovation in hospitals highlights the importance of leadership support and the presence of technologically competent personnel as key enablers of successful adoption and service improvement (Binsar *et al*., 2024). However, resource constraints continue to pose substantial barriers to implementation (Binsar *et al*., 2025a, b).

Digital engagement has been associated with safer and more responsive healthcare services, and it has shown potential in reducing readmissions (Aljafari *et al*., 2024). These developments are mirrored in primary healthcare, where the boundaries between public and private actors are increasingly blurred. In many countries, citizens and private stakeholders actively contribute to the provision of public welfare services, reflecting a shift toward new governance models (Torfing *et al*., 2022; Simonsson *et al*., 2023). This shift is underpinned by the concept of “co-creation”, which refers to the collaborative integration of public and private ideas, resources, and expertise in the development of healthcare services (Torfing *et al*., 2019). In recent years, the term “co-creation” has gained traction in healthcare research (Fusco *et al*., 2023), particularly in the context of digital health innovation. It refers to productive interactions between patients and healthcare professionals where users are engaged as equal partners and active contributors throughout the phases of ideation, design, and testing (den Boer *et al*., 2017; Whitehouse *et al*., 2013). Co-creation is broadly understood as the collective production of innovative outcomes for mutual benefit by various actors (Lipp *et al*., 2023). It is a collaborative process involving providers and consumers, integrating value schemes, shared resources, and mutual learning experiences (Enam *et al*., 2022). In some cases, co-creation extends to direct collaboration between designers or developers and users throughout the entire design process (Mimoso *et al*., 2024). Beyond healthcare, co-creation is increasingly recognised as a new paradigm in public administration. It represents a shift in public service delivery and policy development, promoting a more collaborative and integrated approach (Torfing *et al*., 2019). Unlike traditional coordination or collaboration, co-creation emphasises generating innovative solutions (Krogh *et al*., 2020) and can help ensure that digital health solutions align with end-users’ preferences (Mimoso *et al*., 2024). While some scholars argue that healthcare services are inherently co-created through interactions between providers and recipients (Enam *et al*., 2022), others suggest that co-creation is not automatic and requires active facilitation to occur (Willumsen and Kjeldstadsli, 2020).

However, when addressing complex societal challenges, such as managing rising healthcare demands within limited resources, co-creation often occurs in unstructured environments with diverse and diffuse stakeholders, making facilitation challenging (Torfing *et al*., 2019). Consequently, co-creation does not always emerge from strategic, targeted collaboration but can also arise from spontaneous, uncoordinated actions. The strength of the term is that it “[…] highlights the potential impact of collaborative interaction between public and private actors on the ability to foster new and innovative solutions to intractable problems” (Torfing *et al*., 2019, p. 804). Co-creation thus provides a useful framework for examining interactions between healthcare personnel and suppliers, revealing the diverse roles they play. It serves as an analytical term, acknowledging that actors may not perceive themselves explicitly as co-creators. However, innovative solutions can be constrained when public and private actors fail to adopt roles that enable meaningful engagement in co-creation processes (Torfing *et al*., 2019, 2021; Ansell and Torfing, 2021).

In this article, co-creation is employed as a theoretical lens to explore how collaborative processes involving diverse stakeholders contribute to the transformation of primary healthcare services in Norwegian municipalities. Specifically, the analysis focuses on the implications of technologists’ involvement in co-creation processes and how their involvement influences the development and future direction of primary healthcare services. Rather than approaching co-creation from the perspective of patients – the ultimate end-users – this study focuses on the interactions between institutional actors, such as technologists and municipal healthcare professionals. This focus allows for a deeper understanding of how technological expertise and governance structures shape collaborative interaction and potential innovation in public healthcare settings.

## Methods

### Recruitment and participants

We use the term “welfare technologists” as a collective term that includes representatives of developers and suppliers from private companies. The term “municipal participants” refers to municipal managers or advisors, regardless of education or professional background. Our study sample included 17 participants across 13 interviews (Table 1). Between winter 2021 and spring 2022, and a final interview in spring 2023, we conducted four interviews (three individual and one group interview) with welfare technology companies and nine interviews (six individual and three group interviews) with participants from municipalities. The selected technology companies were key suppliers of welfare technology to Norwegian municipalities, while the municipalities were chosen based on publicly available information about their use of welfare technology. Participants were recruited through direct outreach to both the companies and the municipalities.

**Table 1:** Overview of participants

Company/Municipality	Participant/Role	Quantity	Method	Interview date
GPS company	Business Manager	1	Individual interview	Nov. 2021
Pill dispenser company 1	Consultant	1	Individual interview	Oct. 2021
Pill dispenser company 2	General Manager	1	Individual interview	May 2023
Robotics developer company	Developer	2	Group Interview	Sept. 2021
Municipality A Northern Norway, rural, less populated, long distances	Head of Unit A	1	Individual interview	March 2022
	Head of Unit B	1	Individual interview	March 2022
	Advisor	1	Individual interview	March 2022
	Deputy Manager	1	Individual interview	March 2022
Municipality B Eastern/central Norway, rural, geographically expanded	High-level Manager	1	Individual interview	March 2022
	Team Leader	2	Group Interview	March 2022
	Head of Unit	1	Group Interview	March 2022
	Advisor	1		March 2022
Municipality C Northern/Central Norway, urban, dense population	High-level Manager	1	Individual interview	May 2022
	Mid-level Manager	2	Group Interview	May 2022
Total		17	13	

For the supply side of welfare technology, we conducted four interviews: one individual interview with a business manager from a GPS company, two individual interviews with representatives from pill dispenser producers (a consultant and a general manager), and a group interview with two developers from a robotics developer company.

On the municipality side, we included three municipalities in Norway, selected to ensure variation in geographic location, size, and population density. We identified managers and advisors at the administrative and unit levels through municipal websites and contacted them via e-mail, attaching project information and an invitation to participate. Our purposive sampling focused on managers responsible for the implementation and daily use of welfare technology. Managers were also asked to suggest additional informants at lower levels within their organisations. For managers at the primary care unit level, the roles included both management and nursing tasks, including direct patient contact. All individuals invited to participate accepted the invitation.

### Data collection

The interviews were semi-structured and guided by open-ended questions, allowing participants to emphasise and elaborate on topics they found most relevant to their experiences with welfare technology. Separate interview guides were developed for municipal participants and welfare technologists, each tailored to explore their perspectives on three core areas: (1) collaboration, (2) the responsibilities of technologists, and (3) the impact of increased technology use. These areas were informed by a synthesis of literature on co-creation, particularly within the field of public sector innovation, technology studies, and co-creation theory. This body of work emphasises collaborative processes, shared agency, and the negotiation of roles and responsibilities among stakeholders, and it guided the development of our interview questions.

Examples of questions included: “Who was involved in this process [the procurement], and at what stage?”, “How did the collaboration with the municipality begin, and who or what directed it?”, “What are the expected consequences of this technology (e.g. autonomy, responsibility, dignity, social life, safety, security)?”, “Are there any ethical tensions or dilemmas associated with this technology?”, and “How do you think healthcare services will improve through the use of your technology?” These questions were designed to elicit rich, reflective responses aligned with the three thematic areas and to capture nuanced stakeholder perspectives.

All interviews were conducted via video calls, recorded with participants’ oral consent, and lasted approximately one hour. One author conducted the interviews, while other team members attended and contributed follow-up questions when relevant. All interviews were transcribed verbatim for analysis.

### Data analysis

The data were analysed qualitatively using Braun and Clarke’s (2006, 2022a, b, 2024) reflexive thematic analysis framework, which emphasises the development of meaning-based themes through a process of deep engagement with the data. Our aim was to explore both explicit and implicit meanings related to participants’ experiences with welfare technologies. The analysis began with repeated, immersive readings of the full dataset to ensure familiarity and to begin the process of generating themes and subthemes. All authors independently reviewed the transcripts and contributed to the coding process. Rather than coding data to fit pre-existing themes, we allowed codes to emerge inductively from the data, in line with reflexive TA’s emphasis on researcher subjectivity and interpretative engagement.

As the analysis progressed, we collaboratively developed and refined preliminary themes into broader patterns of shared meaning. These patterns were not treated as pre-existing entities within the data, but as interpretative stories crafted through our analytic lens. To remain close to participants’ lived experiences, we focused on how they described collaboration, roles, responsibilities, and the perceived impact of welfare technologies in healthcare. This process led us to construct three overarching themes: (1) *Welfare technologists enter the public sector arena from below*, (2) *Bridging sectors: The growing complexity of municipality–technologist collaboration*, and (3) *Skill expansion – an impact of emerging technologies*. In line with Braun and Clarke’s conceptualization of themes as meaning-based and multifaceted, each theme represents a central concept with various dimensions that together form a coherent interpretative account of the data. We have analysed the themes in relation to our theoretical framework of co-creation to address our research questions and to illuminate the dynamics and consequences of collaboration between technologists and healthcare professionals. Illustrative quotes from participants are presented in the results section to support and enrich the themes. We found the volume of material manageable and worked closely with the data manually, using only Microsoft Word as our digital tool.

### Ethical considerations

The study was approved by the Office of Research Ethics (id 982,327). Before the interviews, the participants were informed that participation was voluntary and about the study’s details, including anonymity, confidentiality, and their right to withdraw at any time. Informed consent was obtained either via email or orally at the start of the interview.

## Results

Our analysis of the interviews identified three key themes that illustrate collaborative interactions between welfare technologists and healthcare professionals. The following sections will present these themes in detail.

### Welfare technologists enter the public sector arena from below

As outlined in the background section, national authorities have strongly emphasised the need for technological solutions within the healthcare sector. However, welfare technologists rarely cited these directives as their primary motivation for entering the healthcare market. Instead, they seemed to have recognised independently the sector’s escalating challenges and the necessity for targeted measures to sustain satisfactory service delivery. They described the current situation as problematic and urgently requiring intervention. As one participant from a dispenser company put it:

What we saw was that healthcare personnel’s workload today was the same as in 2009 [when the company started]. There were long waiting lists at the GP – what can I say, stressful home care – in other words – primary healthcare. And in hospitals with corridor patients, they struggled. The same picture we see today. And when we started to look more closely at the underlying causes, we saw that the way we treat patients with chronic conditions is very inappropriate. In other words, it is a patient group that accounts for perhaps somewhere between 70–80 per cent of the total resources in our healthcare system. (…) And we said there must be a better way to do this. (General Manager, Pill Dispenser Company 2)

The dispenser supplier reflected on past challenges, noting that conditions in healthcare have not improved significantly since 2009. His retrospective perspective contrasts with another technologist’s forward-looking view, yet both reached the same conclusion: that change is necessary:

We are all familiar with the demographic development, which means that in 2040, in approximately half of Norway’s municipalities, there will be more non-employed people over the age of 67 than employed people aged 20–66. And it’s like – it’s a very drastic change from the current situation. We are in the middle of that avalanche, aren’t we? These demographic trends will force us to work completely differently. (Business Manager, GPS).

As exemplified in the two quotes above, technologists frequently used the pronouns “we” and “us”, emphasising their sense of responsibility and active participation in tackling the challenges within healthcare services. After outlining the current state, a Pill Dispenser producer highlighted the pressing need for improved methods of patient follow-up, underscoring their responsibility to provide viable solutions. The producer elaborated:

We conducted global research to identify regions where health services are provided in a – shall I say, more effective way to follow up with these patients. (…) Early on, we strategically decided to be the best actor in providing users and healthcare personnel with the top software that offers diverse services and enables continuous patient follow-up.

(General Manager, Pill Dispenser Company 2)

This ambition is notable as it addresses the needs of healthcare personnel and patients (or end users). Another technologist echoes a similar patient-centred commitment:

We talk to many people to understand where we can be most helpful. And we see that, especially in home care – although there are other areas within healthcare – many people, for various reasons, struggle to learn new technologies but still benefit immensely from the support that technology can offer (Developer, Robotics Developer Company).

While some welfare technologists expressed a deep commitment to addressing challenges in the healthcare sector, others appeared motivated more by a passion for technology itself. In such cases, one might assume these welfare technologists would pursue opportunities in other industries. However, the healthcare sector’s urgent and unmet needs often draw them in. For instance, robotics developers highlighted their focus on health technology, driven by its pressing demands compared to other sectors. Interestingly, financial success was rarely mentioned as a primary motivation.

Municipal participants strongly emphasised the need for technology, especially in light of demographic changes that are placing immense pressure on healthcare services. Many attributed the implementation of new technology to shortages in medically skilled personnel. One municipal participant described the challenging situation:

This has been our experience over the past two years: dealing with personnel shortages and ensuring that we have enough people available has been challenging. Having the right skills is also challenging. We had to act on the shortage of nurses. (Head of Unit, Municipality A)

The introduction of pill dispensers significantly enhanced staff management of patient care, particularly by optimising their time and resources. One participant explained:

We see the benefit (of the pill dispenser) because they receive their medication at the right time without requiring us to be physically present when they take it (…). This means we can conduct our inspection later in the day. We can manage our time more effectively. Our experience with this technology has been very positive. (Head of Unit, Municipality A)

The statements above illustrate how welfare technologists interviewed were motivated to work with healthcare services by a recognition of the sector’s growing challenges and positioned themselves as solution-oriented partners. Their motivation aligns closely with that of municipal managers. Rather than responding to national directives, implementation of welfare technology is driven by efforts to address systemic challenges through knowledge and understandings acquired from the municipal level.

## Bridging sectors: the growing complexity of municipality-technologist collaboration

Some welfare technologists are directly involved in the development of new technologies, while others function primarily as suppliers, procuring welfare technology from external vendors. However, even welfare technologies acquired externally often require substantial adaptation before it is ready for implementation. The welfare technologists interviewed in this study consistently emphasised that their role extends beyond mere development, sale, or transfer of technology to municipalities. Instead, they highlighted the importance of sustained collaboration with municipal actors throughout the implementation process, highlighting their active involvement in tailoring technological solutions to local needs and contexts. Collaboration was seen as necessary for several reasons. One primary reason is that, although welfare technologies offer numerous benefits, they are not universally applicable. Neither welfare technologists nor healthcare professionals can immediately identify who will benefit most from a given technology. As a result, a collaborative assessment process is needed to ensure appropriate matching between the technology solutions and users’ specific needs and circumstances:

And then we also talk a lot about the fact that the right user may not always be so easy to identify until you have, in a way, tested it out a bit in collaboration – always in collaboration with the user and, in many cases, perhaps almost most, also with relatives. (Consultant, Pill Dispenser Company 2)

The consultant explained that they, in close collaboration with a municipality, accompany healthcare personnel on patient visits to observe the use of their product. By filming and interviewing end users, and involving independent researchers to follow and interview them, they aimed to ensure an unbiased and independent observation and assessment of the product’s functionality concerning the patient’s needs.

However, assessments of patients and product relevance are not always conducted by the welfare technologists themselves, nor is there always direct communication between technologists and the care recipients. Instead, municipal staff are tasked with engaging potential end users and conducting the assessments, although they often require support in determining appropriate candidates for the technology. Welfare technologists viewed the user assessment process as a crucial aspect of training municipal staff, emphasising its importance in ensuring effective technology use:

So, an important part of our training for new municipalities is to go through a user survey, which patients are suitable to use the technology, and which are not? (Consultant, Pill Dispenser Company 2)

In training municipal staff and conducting assessments, technologists presented themselves as “advisors”:

And then I see it at least as my task to be involved in reflecting on it and assist the service out there in reflecting, not telling them how it is, but that they, that we can, reflect together and perhaps ask someone questions that enable them to look at it from a slightly different angle. (Consultant, Pill Dispenser Company 2)

As advisers, they do not focus on selling a product but rather on establishing trust and strengthening municipal staff to face future challenges. Their view that the relationship with the municipalities is one of cooperation rather than a seller-customer relationship became clear. One technologist expressed the perception of a genuinely symmetrical relationship between the technology providers and the municipalities, emphasising that they frequently learn from the municipalities:

Sometimes we must adjust ourselves to the municipalities, and we learn from them and the considerations they take, which we have not thought of. So, there is no doubt that good welfare technology solutions are created when you have put commissioned work and requirement specifications aside. And talk together in a lasting trust-based relationship over time. Then there will immediately be a lot of gold. (Business Manager, GPS Company)

According to the GPS supplier, the supplier and the municipalities must interact to find the best solutions. This interaction requires trust. However, interaction is not always trustworthy, and this way of working together is contrasted with examples of representatives from the municipality administration writing the requirement specifications without involvement from the supplier. The GPS supplier seemed not to trust their competence, as he claimed that they

(…) write a requirement specification about what they think is wise. Perhaps they have been to a few seminars here and there. And then you collaborate – then you communicate around the public marketplace, such as public procurement. That is – that is not why we have good solutions today. (Business Manager, GPS Company)

The GPS supplier described his experience with a seller-customer relationship as unproductive, expressing a preference for a cooperative partnership instead. Another technologist referred to the collaboration among technologists, municipal staff, and end users/next of kin as “tripartite cooperation,” a term with positive connotations in Norwegian. On the other hand, municipalities receive numerous recommendations and inputs from technologists, but not all initiatives are deemed functional. For example, a mapping form for assessing technology needs developed by technologists, albeit in consultation with technology promoters within the municipality, was not found to be effective:

It became a very advanced mapping form that would take up even more of the employees’ time. Like, you had to chart, then you had to check the form; can this user have the technology or not? We concluded that “we scrap the whole thing and distribute these dispensers, and if it doesn’t work, it doesn’t work”. It worked for many. (Head of Unit A, Municipality A)

The fact that municipality A does not always follow technologists’ advice does not necessarily indicate a difficult relationship:

We are quite lucky to have good cooperation with, among others NN (a technologist company), which is very good at technology, you know, but they are trying to sell as much as possible. It is nice to hear what they offer, but I think you have to make qualified decisions for both the municipal economy and other things. There is a lot that is very good and exciting, but you can’t do everything. (Head of Unit A, Municipality A)

As the quote above illustrates, several municipal participants acknowledged the potential benefits of welfare technologies. However, they also expressed awareness that welfare technologists are primarily driven by commercial interests, with a focus on selling products. This awareness was seen as important in shaping the municipalities’ approach to collaboration. One municipal manager, for example, described how they asserted control during a dialogue with a producer, emphasising that the identification of needs and the final decision ultimately rest with the municipality:

After all, we are the ones who decide. So, it doesn’t matter if they try to sell us the products. They are interested in selling us products. We are also interested in establishing contact with them. Because if we find problems that we think can be solved with technology, we want to have a dialogue with them about it. So, I don’t see it as a problem. (Head of Unit B, Municipality A)

One High-level manager (Municipality B) described a need to centralise contact with technology suppliers, assessments of products, and possible procurements. The municipality has entered regional collaborations where the aim is to both ensure knowledge about suppliers and products, and joint agreements and to relieve individual municipalities and individual unit managers of the responsibility of handling offers and approaches from manufacturers and decisions about which products to purchase. The necessity for cross-municipal collaboration was also highlighted in an interview with a mid-level manager (Municipality C). The manager explained that strict procurement rules and agreements necessitate cooperation, facilitated through a cross-municipal forum for purchasing management:

When municipalities begin implementing technology, many take active steps to improve their capacity to manage it independently [without technology suppliers]. This includes hiring technological personnel and fostering cooperation with other municipalities. In Municipality C, a dedicated team established to implement technology into healthcare plays an important role in advancing the municipality’s efforts in this area: They are the driving force. And that means that Municipality C has been involved in many national projects. We also have many, many local projects underway within technology (High-level Manager, Municipality C)

The High-level manager expressed a desire to strengthen the municipality’s competence in implementing technology. This wish appears to stem from a perception that suppliers are overly focused on selling, combined with an awareness that the municipality itself should be responsible for making informed decisions. This has motivated efforts to establish contact and collaboration across municipalities. This section highlights how collaboration between welfare technologists and municipalities is becoming increasingly complex. Technologists advocate for cooperative partnerships and actively contribute to product development, while municipalities emphasise maintaining control over key decisions, particularly in assessing user needs and determining appropriate technological solutions. Municipalities are aware of the tension between commercial interests and the goals of public service. To safeguard public values and retain decision-making authority, they rely on centralised procurement processes and uphold responsibility for care assessments. This balancing act shows a more complex/intricate stakeholder collaboration, where different perspectives are negotiated.

## Skill expansion – an impact of emerging technologies

The interviews revealed a growing consensus among welfare technologists and municipal participants at all levels that technical skills are becoming increasingly needed and important in healthcare. Many welfare technologists expressed confidence that healthcare practices would undergo significant changes driven by the increased demand for technology:

[Home care service] must tune in to a different way of working. They must follow up with patients via a screen and decide who needs follow-up and who can manage on their own. Few are trained in this manner; they are more accustomed to regularly visiting patients in their homes. In many ways, this is no longer the future. (Consultant, Pill Dispenser Company 2)

The welfare technologists were also concerned with the importance of acquiring knowledge or connecting with expertise in the health sector, developing products, establishing good networks, and building good relationships with customers. One participant described how they sought knowledge from health professionals when establishing their company:

First, I realised very quickly that I needed people with a background in healthcare in the entrepreneurial team (…) [to] verify whether the hypotheses we came up with were correct and whether we were going down the right path. (General Manager, Pill Dispenser Company)

Another participant emphasised that their background from working in the health sector helped them understand the need for and see the value of the product they were selling, and that their background contributed to establishing trust in the relationship with the customer:

I wouldn’t have done it if I hadn’t believed in it, and I’ve come to believe in it because I’ve experienced it. And that’s what I want when I’m in dialogue with the municipalities, for them to have confidence in me, for them to understand that I’ve been on their side. I see that it has created value. (Consultant, Pill Dispenser Company)

Emerging welfare technologies are increasingly being used to address local healthcare challenges. As one advisor from the municipalities explained:

We have a lot of faith in home follow-up. That is, since we have 100 km to the hospital. I have a lot of faith in that. Doing digital home follow-up. We have an ongoing wound project. We use a head camera and have the wound outpatient clinic digitally on the eye and ear when we perform wound care. (Advisor, Municipality B)

This quote illustrates how digital solutions are being employed to overcome geographical barriers and improve access to specialised care. The use of remote wound assessment demonstrates growing trust in technology-supported home services and highlights the potential of digital tools to enhance healthcare delivery. Other devices are tailored to specific user needs. For example, passive alert systems are considered more appropriate for people with dementia, who may struggle with active emergency communication. As another advisor explained:

We have now built a new care centre, where we have gathered everyone into one building. And then we have said that in the departments where we have patients with dementia, we have gone for a technology where we have installed a roommate throughout the department, which can provide passive alerts and meet those needs, because then we see that they do not understand this thing about getting a watch on their arm and pressing it if there should be something. (Advisor, Municipality A)

The advisor underscores the municipalities’ role in selecting and implementing welfare technologies that align with the realities of their service users. In addition to implementation, municipalities are investing in spaces that promote learning and awareness around welfare technology. As a deputy manager from one of the municipalities explained:

The Smart House is a display arena for technology, both because I was about to say, so that those who live at home can come and get an intro to what solutions are available. Because it has something to do with starting to mature in relation to the solutions you can apply. We also use it for our own employees for training. Also, it is used for pupils, students, visits from other municipalities, etc. So, it kind of has a pretty wide field then. (Deputy Manager, Municipality A)

The Smart House functions as both a public showroom and a training facility, fostering competence, familiarity, and openness towards assistive technologies. Participants explicitly recognised the potential of technological competence in providing low-threshold support, particularly in contexts where traditional care resources are limited.

One municipal participant described that the staff often expressed that they wanted to learn or upgrade their technical competence, and that they felt they needed to increase their technological competence and skills to feel more secure and to give good care. The manager also described that they now offered more short courses and programs for staff members than before and that they made use of technical experts or IT-support staff for training or support for healthcare workers:

Now we have more and more welfare technology, and we have an increased focus on thorough information. They [staff] can choose different e-learning courses. We have “agents” coming over to our office so they [staff] can ask questions and simply learn more. They [IT staff] can help with issues the staff is struggling with. (Mid-level manager municipality C).

Technical skills and training in these areas are of great significance to health workers in the municipalities. One of the main concerns among the welfare technology suppliers in the study was how to improve the technical skills of both healthcare personnel and patients. Being able to handle and benefit from the technological device largely depends on technical knowledge and skills. In the welfare technologists’ view, technical skills could even substitute for healthcare competence in the absence of skilled staff. One of the municipal participants agreed and emphasised that unskilled health workers could do care tasks if they have enough technical skills:

We depend more and more on help from unskilled workers. (…) Another advantage is that if the user has received a medicine dispenser […] it has to do with the competence of the personnel we send home to that user. Because if there are well-trained unskilled workers, they can do the task of assisting with care and nutrition. Although they have not yet attended medical courses. (Mid-level Manager A, Municipality C)

Unskilled staff who had technical skills were wanted and seen as highly suitable:

High-level Manager, municipality B: It is not certain that you need healthcare personnel for everything, but you do need personnel for everything.

Interviewer: Yes, there are two major challenges you mention there, both with demographics and recruitment. Or access to labour.

High-level Manager, municipality B: Yes. We notice that this is the healthcare workers’ daily situation, I think. By recruiting, I mean the skills and hands needed in the services, it would probably have been tougher without technology.

However, the use of unskilled staff with technical skills to deal with the lack of skilled workers in the health sector was one reason for embracing technology; another reason was to improve the budgets:

We have seen surveys showing that manpower and man-years are saved due to technology. However, using technology also involves cost, so it’s a question of balance, I think. Currently, we have major challenges in recruiting skilled staff; thus, it [technology] is a good solution for handling the situation. (Mid-level Manager, Municipality C).

Even though technical skills and competencies were widely acknowledged as increasingly important within the health sector, municipal participants also expressed concerns about the potential limitations of a technology-driven approach. While they demonstrated an understanding of user capabilities and the need to adapt technological solutions to cognitive and behavioural needs, some questioned whether these technical skills might overshadow the complexity of patients’ lived experiences or even fail, as described by one of the participants from the municipalities:

Before we decide whether to install an alarm, we need to assess the specific need and get consent from the user. […] And that we test that it [the technology] is the (best) alternative before we take it into use. (…) We have decided to be careful so that employees don’t take technology too lightly, and then not make all these assessments.

(Head of Unit B, Municipality A).

These concerns from a head of unit about insufficient assessment or limited familiarity with the technology that could lead to potential failures were particularly pronounced among participants engaged in daily caregiving tasks. Compared to municipal managers, these participants expressed greater hesitation towards expanding the use of technology. They frequently contrasted technological solutions with patients’ individual needs, expressing concerns about the risks of standardising care practices at the expense of personalised care. The theme of “skill expansion” captures a shared understanding among participants that technological skills are becoming increasingly important, not only as a response to workforce shortage, particularly the lack of nurses, but also as part of a broader shift in professional roles and required competencies. Both municipal actors and welfare technologists are developing their skills through mutual learning and collaboration. However, the interviews highlight a tension between technology as efficiency-oriented and healthcare as more value-driven and patient-centred. This tension reveals a concern that the growing emphasis on technological skills may overshadow traditional care values.

## Discussion

In the previous sections, we demonstrated how welfare technologists are increasingly positioning themselves as key actors within the health sector. They not only identify challenges but also propose technological solutions, often in collaboration with municipalities. These partnerships aim to reduce costs and reshape workforce structures. In this section, we discuss these dynamics through the lens of co-creation.

### Technologists as active co-creators in healthcare

Our findings indicate that technologists are not merely responding to predefined demands but actively shaping the development of healthcare services. Drawing on co-creation theories (Torfing *et al*., 2021, 2022; Røiseland and Vabo, 2016; Ansell and Torfing, 2021; Mimoso *et al*., 2024), we conceptualise technologists as active co-creators who participate in defining sectoral challenges and proposing technological solutions. Their framing of issues often aligns with widely accepted “truths” such as demographic changes and the increasing strain on the healthcare sector, which enhances the perceived relevance of their contribution. By offering practical solutions to these challenges, technologists implicitly assume shared responsibility for alleviating pressure on healthcare personnel, thereby strengthening their legitimacy and positioning themselves as essential contributors to healthcare service development. Welfare technologists frequently employ inclusive language, using terms such as “we” when referring to the health sector. This rhetorical strategy positions them as equal stakeholders and aligns their work with overarching goals. However, while this rhetorical strategy fosters a sense of co-creation, it may also obscure underlying commercial interests. As our findings reveal, municipal participants remain aware of the technologists’ dual roles as both salespeople and partners. The procurement of welfare technology is a complex process, involving the identification of specific needs, determination of target groups, selection of suitable technologies, customisation of solutions, and clarification of responsibilities. This complexity underscores the importance of transparent and balanced collaboration. Pojiltov and Mainela (2025) echo this point, emphasising from healthcare innovation networks that effective co-creation depends on understanding and navigating the interplay of diverse and sometimes conflicting goals. Our findings show that co-creation is not a straightforward task, but a dynamic negotiation shaped by differing institutional logics, stakeholder interests, and societal expectations. Recognising this complexity is essential for aligning innovation with broader societal needs and ensuring that co-creation remains both inclusive and accountable.

Reducing this interaction to a simple buyer-seller dynamic would be an oversimplification; rather, it reflects a multifaceted negotiation between stakeholders over healthcare and market values. While power imbalances can hinder co-creation (Torfing *et al*., 2019), actors need not be identical for co-creation to succeed. According to Torvinen and Haukipuro (2017), actors in procurement processes often occupy multiple roles simultaneously. Our findings support this view, showing that welfare technologists frequently act as salespeople, educators, advisors, and suppliers, tailoring technology to both institutional goals and patient needs. Such co-creative practices contribute to innovative solutions to challenges (Torfing *et al*., 2019, 2022; Røiseland and Vabo, 2016) and help ensure that technological implementations align with end-user needs and preferences, a core principle of co-creation (Mimoso *et al*., 2024).

Our findings reveal that both technologists and municipal actors articulate a capacity for mutual learning and the generation of new opportunities and innovations. For example, when nurses transition into technology-related roles or municipalities recruit technology advisors, these cross-disciplinary arrangements foster deeper understanding and help bridge professional divides. This form of cross-boundary learning reflects how co-creation evolves when public and private actors collaborate to address societal challenges (Torfing *et al*., 2021, 192). Such organisational innovations may signal a broader reconfiguration of expertise and responsibilities within municipal care systems, where traditional boundaries between care provision and technological development are increasingly blurred. However, despite these co-creative efforts, tensions persist. Technologists often perceive municipalities as overly cautious or slow-moving, while municipal participants sometimes view technologists as overly eager salespeople who lack sufficient insight into the nuances of municipal care practices. These mismatched expectations can lead to friction and frustration, potentially undermining collaborative processes. Importantly, these tensions do not necessarily indicate failure but rather highlight the fragile and negotiated nature of co-creation, where differing values – commercial versus public services must be continuously aligned.

### Co-creation as a fragile balance

In this study, managers and technologically competent personnel appear to act as key drivers of implementing technology, echoing patterns observed in hospital settings (Binsar *et al*., 2025a, b) and healthcare networks (Pojiltov and Mainela, 2025). High-level municipal managers, often aligned with national welfare technology policies, tend to share perspectives with technology suppliers. They enthusiastically embrace welfare technology, highlighting their comprehensive efforts to integrate devices such as pill dispensers and personal security alarms into municipal health services. These technologies are perceived as essential, desirable, and easy to implement. Technological expertise is highly valued, symbolising innovation and progress, while a lack of digital skills is increasingly viewed as outdated.

**Figure 1:**
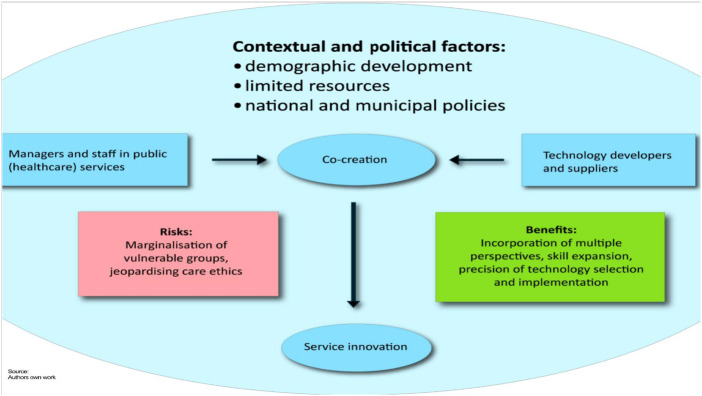
A visual framework of co-creation processes. Source: Authors’ own work

While national policies encourage the adoption of technology, final decisions seem to rest with local actors. This autonomy allows municipalities to act entrepreneurially (Lipp *et al*., 2023) while ensuring that technology adoption aligns with genuine needs (Gjerstad *et al*., 2025). A central theme emerging from our data is the strategic role of municipalities in co-creation processes. Municipalities retain control over procurement and evaluation to ensure that technological solutions reflect public values. This control is not merely procedural but serves as a safeguard against the risks of outsourcing, allowing municipalities to shape the terms of the collaboration. While power hierarchies among professionals, patients, and stakeholders are well-documented (Singh, 2017), our findings suggest that co-creation can foster balanced partnerships. The co-creation processes in this study appear to be consensual, enabling stakeholders to work toward shared goals. As our findings show, there appears to be a balance between technologists and municipalities, forming the foundation for genuine co-creation. Co-creation is widely recognised as a collaborative process among diverse actors to address societal challenges (Torfing *et al*., 2019). A foundation of mutual respect and equal participation fosters productive collaboration, yielding benefits for all involved (Røiseland and Vabo, 2016; Lipp *et al*., 2023). However, public debates highlight concerns that technologists may be “colonising” healthcare services rather than co-creating and collaborating. Many municipal action plans explicitly promote welfare technology implementation, granting technologists a strategic advantage. National and local advocacy for increased technology use further legitimises their role, reinforcing their influence. The language of technological optimism, embedded in policy documents, risks marginalising dissenting perspectives (Pollock and Williams, 2009). There is also a broader concern that private companies may dominate the development, ownership, and governance of welfare technologies. Such dominance could “[…] pave the way towards a hidden privatization of (future) services and a re-allocation of control away from citizens” (Lember *et al*., 2019, p. 1682), ultimately affecting healthcare professionals’ care ethical decision-making (see Figure 1). While the relationship between municipalities and technologists fosters innovation and is characterised by co-creative processes, it can also be understood as a fragile relationship.

## Strengths and limitations

While this study offers valuable insights into the dynamics of co-creation in the implementation of welfare technology within Norwegian municipalities, certain limitations should be acknowledged.

One notable limitation is the exclusive focus on the provider side of healthcare services, omitting the perspective of patients and their relatives. This focus is justified by the underexplored role of technologists in health services research, despite their crucial influence on the valuation and implementation of technology. The participant selection process, which involved managers recommending additional participants, ensured relevance but may have introduced bias based on managerial attitudes. However, the data does not indicate significant bias, as participants expressed both positive and negative perspectives.

A key strength of this study lies in its dual perspective, incorporating insights from both private-sector technologists and public healthcare managers and professionals. The analysis highlights the complexity of integrating welfare technology into primary healthcare systems. By employing a co-creation framework, the study sheds light on the distinct roles of co-creators and offers valuable insights into how these actors navigate the intersection of care work and technological development.

## Concluding remarks

This study explores how welfare technologists have positioned themselves as key actors in the primary healthcare sector. Using theories of co-creation, we analysed their engagement in developing healthcare services. The study reveals that welfare technologists strategically present themselves as well-informed about the sector’s challenges. This enables them to gain acceptance within municipalities, where they are recognised as important partners in shaping healthcare services.

Our findings highlight that implementing welfare technology in primary healthcare services requires significant communication and collaboration between welfare technologists and municipal staff. Both groups have begun integrating expertise from the other side: technology companies hire nurses, and municipalities employ technology advisors. This exchange of expertise not only facilitates smoother collaboration but also enhances the precision of technology selection and implementation. Looking ahead, both welfare technologists and municipalities envision a growing role for technologically skilled personnel within healthcare. These personnel would take on direct responsibilities for managing technology, reducing reliance on traditional nursing roles, and enabling municipalities to reorganise their workforce more efficiently.

Co-creation offers a promising avenue for amplifying participation by incorporating multiple actors and perspectives, ideally leading to more democratic processes. However, there is a risk that the voices of vulnerable groups may be overlooked or marginalised in these multi-actor settings. Although municipalities can strengthen their negotiating position and improve procurement practices, the increased influence of welfare technology and welfare technologists could unintentionally make it harder for end users to effectively communicate their needs and preferences. The increasing role of welfare technologists in healthcare raises important questions about control, autonomy, and the future governance of welfare technologies. It may reflect a broader shift in healthcare governance, where healthcare services are co-constructed through cooperation, mutual learning, and the balancing of technical possibilities with care values. Recognising and managing these tensions is therefore crucial for sustaining meaningful collaboration and ensuring that technological innovations are both relevant and ethically grounded within the context of municipal care.

We believe our findings provide valuable insights into co-creating processes in healthcare, particularly as they bridge two interconnected but structurally distinct domains of welfare provision. Future research should delve deeper into these dynamics, focusing on balancing technological advancement with patient-centred care and identifying pathways for fostering more equitable and effective partnerships between stakeholders in healthcare.

## Data Availability

The data will be available as anonymised transcripts from the corresponding author upon reasonable request.
